# FliO Regulation of FliP in the Formation of the *Salmonella enterica* Flagellum

**DOI:** 10.1371/journal.pgen.1001143

**Published:** 2010-09-30

**Authors:** Clive S. Barker, Irina V. Meshcheryakova, Alla S. Kostyukova, Fadel A. Samatey

**Affiliations:** 1 Trans-Membrane Trafficking Unit, Okinawa Institute of Science and Technology, Okinawa, Japan; 2 Department of Neuroscience and Cell Biology, Robert Wood Johnson Medical School, Piscataway, New Jersey, United States of America; Washington University School of Medicine, United States of America

## Abstract

The type III secretion system of the *Salmonella* flagellum consists of 6 integral membrane proteins: FlhA, FlhB, FliO, FliP, FliQ, and FliR. However, in some other type III secretion systems, a homologue of FliO is apparently absent, suggesting it has a specialized role. Deleting the *fliO* gene from the chromosome of a motile strain of *Salmonella* resulted in a drastic decrease of motility. Incubation of the Δ*fliO* mutant strain in motility agar, gave rise to pseudorevertants containing extragenic bypass mutations in FliP at positions R143H or F190L. Using membrane topology prediction programs, and alkaline phosphatase or GFPuv chimeric protein fusions into the FliO protein, we demonstrated that FliO is bitopic with its N-terminus in the periplasm and C-terminus in the cytoplasm. Truncation analysis of FliO demonstrated that overexpression of FliO_43–125_ or FliO_1–95_ was able to rescue motility of the Δ*fliO* mutant. Further, residue leucine 91 in the cytoplasmic domain was identified to be important for function. Based on secondary structure prediction, the cytoplasmic domain, FliO_43–125_, should contain beta-structure and alpha-helices. FliO_43–125_-Ala was purified and studied using circular dichroism spectroscopy; however, this domain was disordered, and its structure was a mixture of beta-sheet and random coil. Coexpression of full-length FliO with FliP increased expression levels of FliP, but coexpression with the cytoplasmic domain of FliO did not enhance FliP expression levels. Overexpression of the cytoplasmic domain of FliO further rescued motility of strains deleted for the *fliO* gene expressing bypass mutations in FliP. These results suggest FliO maintains FliP stability through transmembrane domain interaction. The results also demonstrate that the cytoplasmic domain of FliO has functionality, and it presumably becomes structured while interacting with its binding partners.

## Author Summary

The propeller-like flagella, which some bacteria use to swim, possess a specialized secretion apparatus, which is imbedded in the cell membrane for their formation. The components are highly conserved among flagella systems and also to the Type III secretion apparatus used by some bacteria in conjunction with virulence-associated needle complexes. The ubiquity of these secretion apparatuses and their function as intricate nanomachines has made them fascinating for biologists. The most studied flagellar system is that of *Salmonella enterica*, which consists of 6 integral membrane proteins: FlhA, FlhB, FliO, FliP, FliQ, and FliR. Among these proteins, FliO shows a sporadic distribution in bacteria, and its function is unknown, suggesting it might have a specialized role to play where it is present. In this study, we show that FliO has an important role in maintaining stability of FliP, which is a highly conserved member of the secretion apparatus. We have characterized the important regions of FliO through mutagenesis. We have shown that it is possible to bypass the effect of not producing the FliO protein, by encoding mutations within FliP or by overexpressing the cytoplasmic domain of FliO only.

## Introduction

For many bacteria, locomotion is possible using the flagellum, which functions like a helical propeller. It is a highly complex nanomachine consisting of about 30 different proteins, and it is organized into three substructures: the basal body, the hook, and the filament. Export of the components of the flagellum across the cytoplasmic membrane requires a specialized secretion apparatus at its base, which shares homology to the type III secretion apparatus of the bacterial needle used by some Gram-negative bacteria in pathogenesis [1]. For the flagella systems of *Escherichia coli* and *Salmonella enterica* serovar Typhimurium, the secretion apparatus is postulated to consist of six integral membrane proteins: FlhA, FlhB, FliO, FliP, FliQ, and FliR; and three cytoplasmic proteins: FliH, FliI and FliJ [2], [3]. Biochemical and genetic studies have determined the location of the membrane proteins FlhA, FlhB, FliP, and FliR to be within the flagellar basal body [4]–[6]. It has been demonstrated that the hook-capping protein (FlgD) and hook protein (FlgE) required all of the proteins of the secretion apparatus for their export [7].

A detailed picture of the workings of the secretion apparatus is gradually being elucidated [8], [9]. It has been demonstrated that the secretion apparatus harnesses the proton motive force to drive export of the external flagellar components [10], [11]. FlhA and FlhB, the two largest membrane proteins of the flagellar secretion apparatus, which both have predominant C-terminal cytoplasmic domains have been most characterized. The crystal structures of the cytoplasmic domains of FlhA from *Salmonella* and *Helicobacter pylori*, and the crystal structure of the cytoplasmic domain of the type III secretion system apparatus paralogue InvA from *Salmonella* have recently been solved [12]–[14]. Also, several crystal structures of the cytoplasmic domains of paralogues of FlhB found in virulence-associated needles from enteric bacteria have recently been described [15]–[17].

The cytoplasmic domains of FlhA and FlhB form a docking platform for FliH, FliI, and FliJ. The cytoplasmic domain of FlhA has been shown to bind the FliH, FliI, and FliJ proteins, and it is also thought to be directly involved in the translocation of the export substrates into the central channel of the growing flagellar structure [18]–[22]. The cytoplasmic domain of FlhB has been shown to undergo autocleavage associated with interaction with FliK, which switches specificity of export of rod/hook-like substrates to filament-type substrates [23]–[29]. The N-terminal transmembrane region of FlhA has been implicated to interact with the surrounding MS ring, from studies involving the isolation of extragenic suppressor mutations [5].

Less is known about the functional role of the FliO, FliP, FliQ, and FliR proteins in the secretion complex, though the protein products of the corresponding genes have been determined [30]–[33]. FliP, FliQ, and FliR are very hydrophobic and were predicted to be predominantly located within the cytoplasmic membrane. However, FliO was predicted to be a bitopic membrane protein with a predominant soluble domain. Intriguingly, FliO shows the least conservation among the secretion system apparatus membrane proteins, even being absent from some systems [3], [34], [35]. Notably, the type III secretion system apparatus of the virulence-associated needles of *Salmonella* serovar Typhimurium lack a FliO homologue, as does the flagellum of the ancient hyper-thermophilic bacterium *Aquifex aeolicus* [3], [35]. Since the FliO protein is not necessary for some systems it suggests that the FliO protein might function as an accessory protein and have a specialized role where it is present. We have undertaken a mutational analysis of FliO described herein, to determine whether or not it is essential for flagellar assembly, which parts of the protein are important, its orientation in the membrane, and possible interaction partners to help understand its function.

## Results

### Incubation of a Δ*fliO* mutant in motility agar gave rise to pseudorevertants containing bypass mutations in *fliP*

**Figure 1:**
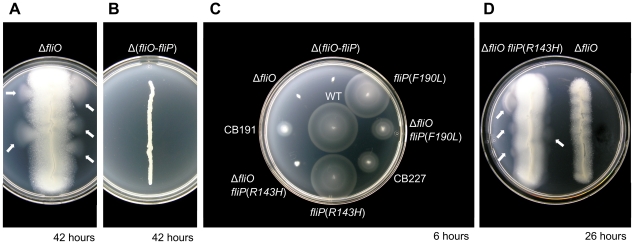
Isolation of pseudorevertants containing bypass mutations in *fliP* from a poorly motile Δ*fliO* mutant. (A) Incubation of a Δ*fliO* strain, CB186, in soft-tryptone motility agar after 42 hours. The strain is extremely poorly motile, but outgrowths of motile pseudorevertants can be observed as motility halos, indicated by white arrows. (B) Incubation of a non-motile Δ(*fliO*-*fliP*) strain, CB184, in motility agar after 42 hours. (C) Comparison of motility of pseudorevertants, purified from CB186, to engineered strains after 6 hours in motility agar: WT, wild-type is strain SJW1103; CB191 is a pseudorevertant strain and encodes Δ*fliO fliP*(*R143H*); CB227 is a pseudorevertant strain and encodes Δ*fliO fliP*(*F190L*). Other strains were engineered mutants constructed from SJW1103 by *λ*-Red genetic engineering. (D) Formation of motile pseudorevertants by the Δ*fliO fliP*(*R143H* ) engineered mutant after only 26 hours. All plates were incubated at 30°C.

We first sought to characterize the FliO protein through mutagenesis to investigate its function. To provide a genetic background for complementation studies, a Δ*fliO* mutant was engineered from the wild-type strain SJW1103. We found that the Δ*fliO* mutant, CB186, was not completely non-motile, and displayed a weakly motile phenotype after several hours in motility agar. Surprisingly, pseudorevertants arose from this strain with enhanced motility after about 42 hours extended incubation (Figure 1A). In comparison strain CB184, which encodes a non-polar Δ(*fliO-fliP*) deletion was completely non-motile (Figure 1B). Four motile pseudorevertants from CB186 were purified, and the two most motile were characterized further. These two strains were designated CB191 and CB227.

To screen for the bypass mutations the genomic DNA of CB191 and CB227 was isolated and the *flhA, flhB, fliO, fliP, fliQ*, and *fliR* genes were sequenced along with the *fliF* gene encoding the MS ring, which surrounds the secretion apparatus in the membrane. These genes were considered the most likely to contain suppressor mutations. Each pseudorevertant contained a point mutation in the *fliP* gene only. CB191 encoded an R143H mutation in FliP, and CB227 encoded an F190L mutation in FliP.

To investigate the physiological effects of the extragenic bypass mutations in *fliP*, engineered strains were constructed from SJW1103 using *λ*-Red genetic engineering (Figure 1C). Engineered mutants encoding *fliP*(R143H) or *fliP*(F190L) mutations only showed the same motility phenotype as SJW1103. Moreover, an engineered mutant encoding Δ*fliO fliP*(*F190L*) was equally as motile as CB227, which confirmed that the *fliP*(*F190L*) mutation was responsible for the extragenic suppression of the *fliO* deletion in strain CB227. However, an engineered strain encoding Δ*fliO fliP*(*R143H*) was not as motile as CB191. This appeared to suggest that the *fliP*(*R143H*) mutation was not the suppressor mutation responsible for the improved motility in pseudorevertant strain CB191. However, the engineered Δ*fliO fliP*(*R143H*) mutant gave rise to pseudorevertants with enhanced motility after a much shorter incubation time of 26 hours in motility agar in comparison to a Δ*fliO* mutant, which took at least 42 hours (Figure 1D). Therefore, in strain CB191 an additional bypass mutation must be encoded along with *fliP*(*R143H*) to overcome the Δ*fliO* deletion.

### The FliO N-terminus is in the periplasm and its C-terminus is in the cytoplasm

The above results suggested that FliO is regulating FliP so we decided to characterize the FliO protein further to determine how this might occur. Prior to this study the membrane topology of FliO was unknown, so we started by determining the topology of FliO. We used various prediction programs for the determination of the topology of transmembrane proteins, and the majority suggested that the 125-residue FliO protein is bitopic with a short N-terminal periplasmic domain from residues 1 to 16–22, a transmembrane region between residues 17–23 to 39–43, and a large C-terminal cytoplasmic domain from residues 40–44 to 125 (Table S1).

**Figure 2:**
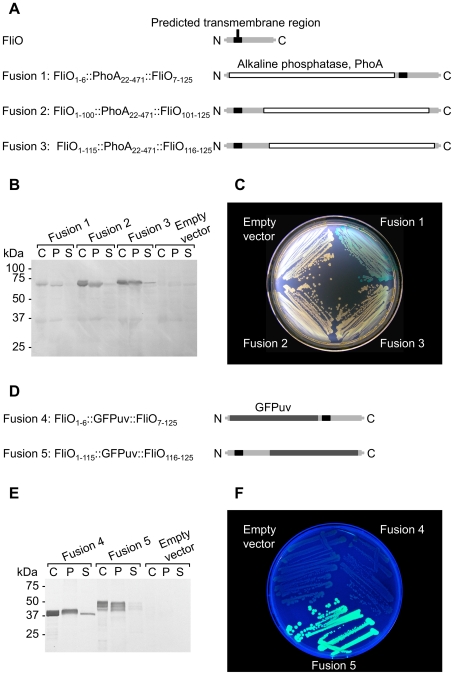
The FliO N-terminus is in the periplasm and its C-terminus is in the cytoplasm. (A) Schematic overview of chimeric fusions of alkaline phosphatase within FliO. (B) Immunoblotting using anti-alkaline phosphatase antibody against cell fractions of strains expressing FliO/alkaline phosphatase chimeras (60.3 kDa): C, whole cells; P, insoluble membrane pellet fraction; S, soluble supernatant fraction. (C) Growth of strains expressing FliO/alkaline phosphatase chimeras on L-agar plates containing 40 µg ml^−1^ 5-Bromo-4-chloro-3-indoyl phosphate at 37°C. All strains contained a Δ*phoN301* deletion. (D) Schematic overview of chimeric fusions of GFPuv within FliO. (E) Immunoblotting using anti-GFP antibody against cell fractions of strains expressing FliO/GFPuv chimeras (39.9 kDa). (F) Growth of strains expressing FliO/GFPuv chimeras on L-agar plates at 30°C. All fusions were expressed from plasmid pTrc99A-FF4 without IPTG induction.

To experimentally determine the membrane topology of FliO we constructed plasmids to express chimeric gene fusions of the *phoA* gene encoding the mature form of alkaline phosphatase, at three specific points into the *fliO* gene. The mature form of alkaline phosphatase is only active when found in the periplasm, and its use is well established to determine membrane topology of transmembrane proteins [36]–[38]. The entire amino acid sequence of FliO was present in fusion product; and alkaline phosphatase was encoded between residues 6 and 7, 100 and 101, or 115 and 116 of FliO (Figure 2A). These fusion sites were permissive for motility (data not shown). Immunoblotting using anti-alkaline phosphatase antibody against whole cell lysates of cells expressing the fusions, and the lysates fractioned into insoluble membrane pellet fractions and soluble supernatant fractions demonstrated that the three FliO/PhoA chimeras were expressed stably and mainly partitioned with the insoluble membrane pellet fraction (Figure 2B). To detect expression of alkaline phosphatase, cells expressing the plasmid encoded fusions were inoculated onto L-agar plates containing 40 µg ml^−1^ 5-Bromo-4-chloro-3-indoyl phosphate (Figure 2C). The *phoN* gene was deleted from the chromosome of the strains used in this experiment, to reduce background phosphatase expression. Alkaline phosphatase activity was detectable only with the FliO_1–6_::PhoA_22–471_::FliO_7–125_ fusion. These fusions were next genetically engineered onto the chromosome of *Salmonella*. Alkaline phosphatase assays were performed, and higher phosphatase activity was measured for the FliO_1–6_::PhoA_22–471_::FliO_7–125_ fusion, compared to the other chimeras, whether it was expressed from the chromosome only or also expressed from plasmid pTrc99A-FF4 (Table S2). This revealed that the N-terminus of FliO is in the periplasm.

GFPuv has been reported to be useful as a reporter for membrane protein topology in bacteria and it is fluorescent when it is located in the cytoplasm [39]. We constructed plasmids to encode chimeric gene fusions of the GFPuv gene between residues 6 and 7, or 115 and 116 of FliO (Figure 2D). These fusions were permissive for motility (data not shown). Immunoblotting using anti-GFP antibody against whole cell lysates of cells expressing the fusions, and the lysates fractioned into insoluble membrane pellet fractions and soluble supernatant fractions demonstrated that the two FliO/GFPuv chimeras were expressed stably and mainly partitioned with the insoluble membrane pellet fraction. However, a difference in migration between the two fusions is apparent, and the FliO_1–115_::GFPuv::FliO_116–125_ fusion produced multiple bands (Figure 2E). The reason for this is not clear. In previous studies, multiple bands of FliO were detected by immunoblotting [32], [33]. The authors could not conclusively identify the source of the multiple bands, but speculated that the N-terminus of FliO underwent proteolytic cleavage, which did not appear to be physiologically important. We presume that the chimeric fusions we have constructed are somehow affecting this N-terminal modification of FliO. However, fluorescence was detectable only with the FliO_1–115_::GFPuv::FliO_116–125_ fusion (Figure 2F). This revealed that the C-terminus of FliO is in the cytoplasm.

### Mutational analysis of FliO reveals the cytoplasmic domain of FliO is functionally important

**Figure 3:**
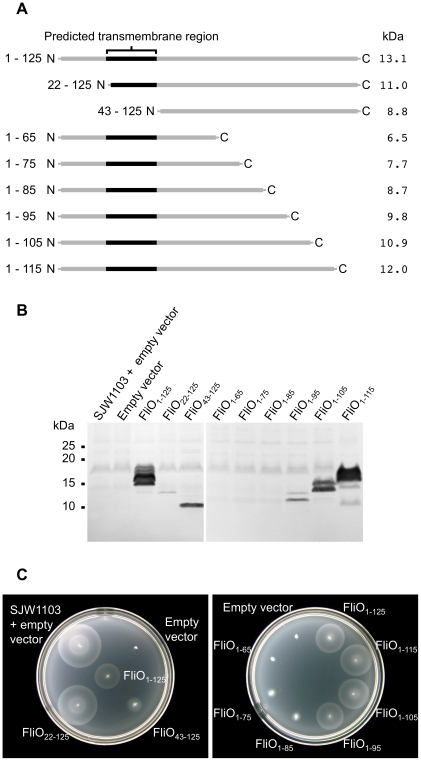
Complementation of a Δ*fliO* strain by FliO truncated at the N-terminus or C-terminus. (A) Schematic overview of FliO deletion mutants with the corresponding molecular weight of the protein. (B) Immunoblotting using polyclonal anti-FliO_43-125_-6xHis antibody against 10–20% gradient SDS-PAGE separated whole cell lysates of the wild-type strain, SJW1103, containing empty pTrc99A-FF4 vector, or a Δ*fliO* mutant (strain CB186) containing empty vector, or strain CB186 expressing the truncated FliO proteins from plasmid pTrc99A-FF4 (without IPTG induction). (C) Complementation of strain CB186 by the truncated FliO proteins expressed from plasmid pTrc99A-FF4. SJW1103, containing empty pTrc99A-FF4 vector, was included for comparison. Soft-tryptone motility agar inoculated with the strains was incubated for 6 hours at 30°C.

Having defined the membrane topology of FliO, we next sought to determine the functionally important regions of the FliO protein by examining the ability of FliO proteins truncated from the N-terminus or the C-terminus to rescue the motility of a Δ*fliO* strain in motility agar. The truncated proteins were expressed from plasmid pTrc99A-FF4 without IPTG induction (Figure 3A). Immunoblotting using polyclonal anti-FliO_43–125_-6xHis antibodies was performed first to confirm expression of the proteins. Full-length FliO_1–125_, FliO_22–125_, FliO_43–125_, FliO_1–95_, FliO_1–105_, and FliO_1–115_ were detected by immunoblotting (Figure 3B). FliO_1–125_, FliO_1–95_, FliO_1–105_, and FliO_1–115_ produced multiple bands of FliO, while FliO_22–125_ and FliO_43–125_ did not. As mentioned in the previous section, it was suggested in a previous study that FliO is subject to N-terminal cleavage or modification by an unknown mechanism and the multiple bands presumably reflect this [33]. It was not possible to detect FliO at physiological levels from whole-cell lysates of SJW1103, therefore, the proteins produced from pTrc99A-FF4 were detectable by immunoblotting, because they were being over-expressed from this vector. It was not possible to detect FliO_1–65_, FliO_1–75_, and FliO_1–85_ by immunoblotting, which could be due to the specificity of the antibody or because these proteins were not expressed. A FLAG-tag (N-MDYKDDDDK-C) was engineered at the N-terminus of FliO_1–125_, FliO_1–65_, FliO_1–75_, and FliO_1–85_ to attempt to detect protein expression by immunoblotting using ANTI-FLAG antibody. However, only FLAG-tagged FliO_1–125_ was detectable by immunoblotting, so if FLAG-tagged FliO_1–65_, FliO_1–75_ and FliO_1–85_ were produced expression was much lower (data not shown).

Expression of FliO_22–125_ or FliO_43–125_, which are truncated at the N-terminus, was able to complement the Δ*fliO* strain CB186 (Figure 3C). FliO_22–125_ encoded a methionine at position 22 in place of a natural valine and this truncation was included since it has been shown to be functional in previous studies [31], . Surprisingly, FliO_43–125_ could complement, as this corresponds to the cytoplasmic domain of FliO, and is without the transmembrane domain. Overexpression was necessary for the cytoplasmic domain to rescue motility of CB186, since FliO_43–125_ was not able to complement when expressed from the T7 promoter of plasmid pET-22b(+), without IPTG induction, while full-length FliO_1–125_ was able to rescue motility when expressed from this vector (data not shown). FliO_1–95_, FliO_1–105_, and FliO_1–115_, which are truncated from the C-terminus were able to restore almost full motility to CB186, so the C-terminal 30 amino acids of FliO are not essential. In comparison, FliO_1–75_ and FliO_1–85_ could only weakly restore motility to CB186, while FliO_1–65_ could not (Figure 3C).

### Residue leucine 91 of the cytoplasmic domain of FliO is important for function

**Figure 4:**
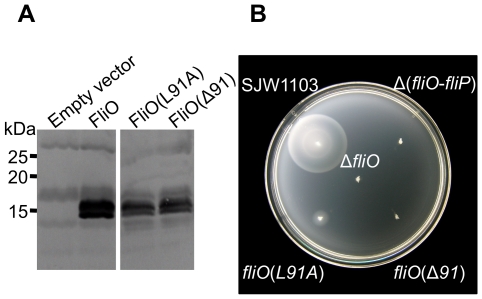
Residue leucine 91 is important for FliO function. (A) Immunoblotting using polyclonal anti-FliO_43–125_-6xHis antibody against 10–20% gradient SDS-PAGE separated whole cell lysates of a Δ*fliO* mutant (strain CB186) containing empty vector, or strain CB186 expressing FliO or mutated FliO proteins (approximately 13 kDa) from plasmid pTrc99A-FF4 without IPTG induction. (B) Incubation of the wild-type strain SJW1103 or genetically engineered mutants in soft-tryptone motility agar for 6 hours at 30°C.

N-terminal and C-terminal truncation analysis of FliO have defined residues 22–95 as the most important. Moreover, overexpression of the cytoplasmic domain of FliO could rescue motility of a Δ*fliO* mutant, suggesting that this domain is functionally important. We next undertook a site-directed mutagenesis study of the cytoplasmic domain to identify important residues to confirm it is functionally important. We aligned the FliO proteins from closely related *Gammaproteobacteria*, to identify conserved residues. There was considerable sequence variation, but several conserved amino acids between residues 43–95 of the cytoplasmic domain of FliO could be identified (Figure S1). To screen for the important residues, we performed site-directed mutagenesis for full-length FliO creating point substitutions for alanine or point deletions at the following positions: G64, R68, V74, G82, T84, L91, and L94. Plasmids encoding these mutations were then examined for their ability to rescue motility of the Δ*fliO* mutant, CB186, in soft-tryptone motility agar. FliO(Δ91) was identified to cause a reduction in motility (data not shown). Immunoblotting using polyclonal anti-FliO_43–125_-6xHis antibodies demonstrated that FliO(L91A) and FliO(Δ91) could be expressed stably (Figure 4A). Strains containing the *fliO*(*L91A*) or *fliO*(Δ*L91*) mutant alleles on the chromosome were engineered, and both strains were dramatically less motile (Figure 4B). Residue leucine 91 of the cytoplasmic domain is therefore very important for the function of FliO.

### Circular dichroism (CD) spectroscopy analysis of the cytoplasmic domain of FliO

**Figure 5:**
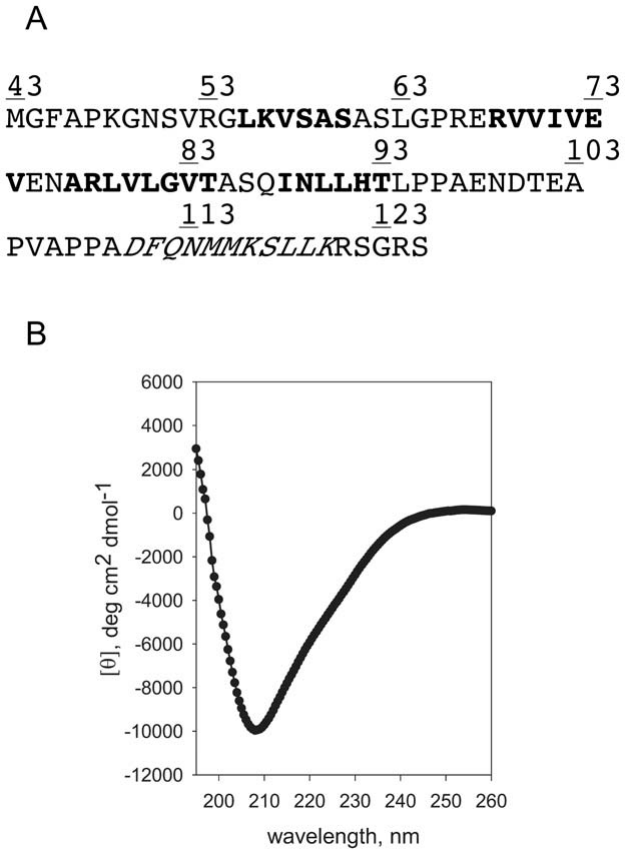
Circular Dichroism (CD) Spectroscopy analysis of the cytoplasmic domain of FliO. (A) Predicted regions of secondary structure of FliO_43–125_: Bold, regions with predicted *β*-structure; Italic, regions with predicted *α*-helix. (B) CD spectrum (mean residue ellipticity vs wavelength) of FliO_43–125_-Ala in 20 mM Na/K phosphate, pH 6.2; 100 mM NaCl.

We have revealed that the cytoplasmic domain of FliO is functionally important, so we next studied this domain by circular dichroism (CD) spectroscopy to see whether it is structured. Based on secondary structure prediction the cytoplasmic domain of FliO should contain four beta-strands and one alpha-helix (Figure 5A). FliO_43–125_-Ala was purified using a C-terminal intein fusion tag. An additional alanine residue at the C-terminus was added to facilitate removal of the intein-tag. After cleavage of the intein fusion tag and purification FliO_43–125_-Ala was studied using CD spectroscopy. From the shape of the spectra, the structure of FliO_43–125_-Ala apparently consists mostly of random coil and some *β*-structure (Figure 5B). Melting of FliO_43–125_-Ala was irreversible due to aggregation, this often happens with *β*-structural proteins [40]. Then FliO_43–125_-Ala was titrated with urea and no two-state transition was observed. From these data we conclude that this peptide is disordered and its structure is a mixture of random coil and beta-sheet.

### Full-length FliO increases FliP expression

**Figure 6:**
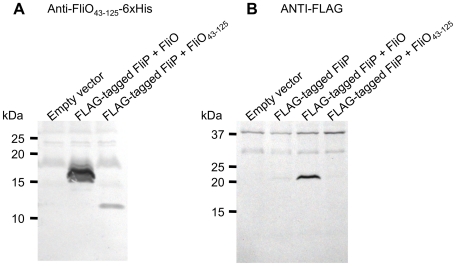
FliO increases expression of FliP. FLAG-tagged FliP (25.4 kDa) was expressed alone, or co-expressed with FliO (13.1 kDa), or co-expressed with the cytoplasmic domain of FliO, FliO_43–125_ (8.8 kDa), from plasmid pTrc99A-FF4 without IPTG induction. Whole cell lysates were separated by 10–20% gradient SDS-PAGE, prior to immunoblotting. (A) Immunoblotting with polyclonal anti-FliO_43–125_-6xHis antibody. (B) Immunoblotting with ANTI-FLAG antibody. Results are representative of the experiment performed in triplicate.

We have shown that bypass mutations in *fliP* can rescue motility for cells deleted for the *fliO* gene. We have also shown through mutagenesis that the most important residues of FliO are between amino acids 22 to 95, and the cytoplasmic domain alone has functionality. To further characterize the apparent regulation of FliP by FliO, we investigated the effect of FliO co-expression on the synthesis of plasmid expressed FLAG-tagged FliP in the Δ(*fliO-fliP*) strain CB184. A FLAG-tag epitope (N-DYKDDDDK-C) was encoded between codons 22 and 23 of *fliP*. The FLAG-tag was added to enable detection of FliP expression by immunoblotting with ANTI-FLAG antibody. After cleavage of the N-terminal signal peptide of FliP, which occurs between residues 21 and 22, the FLAG-tag would be encoded immediately after the glutamine residue. FLAG-tagged FliP was expressed alone, or co-expressed with full-length FliO, or co-expressed with the cytoplasmic domain of FliO. FLAG-tagged FliP expression levels were not improved if the R143H or F190L bypass mutations were encoded (data not shown). Expression of full-length FliO or the cytoplasmic domain FliO_43–125_, was detected from the co-expression vectors by immunoblotting with polyclonal anti-FliO_43–125_-6xHis antibody (Figure 6A). Expression of FLAG-tagged FliP was dramatically improved when full-length FliO was co-expressed. However, expression levels of FLAG-tagged FliP were not improved when the cytoplasmic domain of FliO was co-expressed (Figure 6B).

### Overexpression of the cytoplasmic domain of FliO in strains deleted for *fliO* and expressing bypass mutations in *fliP* further rescues motility

**Figure 7:**
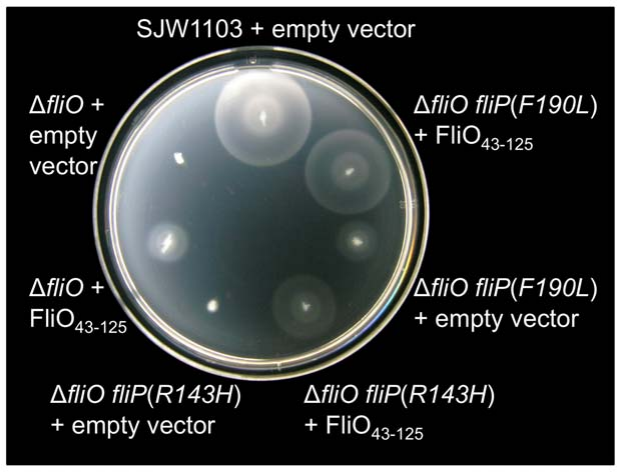
Rescue of motility by overexpression of the cytoplasmic domain of FliO. SJW1103 is a wild-type strain. Strains genetically engineered from SJW1103 contained a *fliO* gene deletion with or without bypass mutations in the gene for *fliP*. The cytoplasmic domain of FliO, FliO_43–125_, was expressed from plasmid pTrc99A-FF4 without IPTG induction. Soft-tryptone motility agar inoculated with the strains was incubated for 6 hours at 30°C.

We have revealed that full-length FliO can stabilize FliP expression, while the cytoplasmic domain of FliO cannot. We have also shown that overexpression of the cytoplasmic domain of FliO, FliO_43–125_, can rescue motility of cells with the *fliO* gene deleted. We have further shown that extragenic bypass mutations within the *fliP* gene can also partially restore motility to the Δ*fliO* mutant. We next demonstrated that expression of FliO_43–125_ from pTrc99A-FF4 could further rescue motility to near wild-type levels for engineered strains encoding the Δ*fliO fliP*(*R143H*) or Δ*fliO fliP*(*F190L*) mutations (Figure 7).

## Discussion

In this study, we have undertaken a detailed molecular analysis of the FliO secretion apparatus protein and its role in flagella assembly. FliO was previously considered to be necessary for secretion in *Salmonella* [7]. However, we have demonstrated using an engineered non-polar Δ*fliO* mutant that while deletion of the *fliO* gene leads to a dramatic reduction in motility, cells are not completely non-motile. Moreover, it was possible to readily isolate pseudorevertants containing bypass mutations in *fliP*, which help to rescue motility. The FliO protein is not conserved in all type III secretion systems, yet in this study we have shown that it has an important functional role in regulating FliP stability, which is a highly conserved member of the secretion system membrane proteins.

We have also shown that FliO has a predominant C-terminal cytoplasmic domain, which is in contrast to the previously predicted membrane topology of FliO, which suggested that the C-terminus is in the periplasm [32]. Coexpression of the cytoplasmic domain of FliO with FliP did not increase FliP expression levels, while coexpression of full-length FliO with FliP improved FliP expression levels. This suggests that the transmembrane region of FliO stabilizes FliP, since the periplasmic domain of FliO appears to be non-essential from our truncation analyses. However, we have shown that the cytoplasmic domain represents a functional unit beyond merely being required for FliO membrane insertion, since overexpression of the cytoplasmic domain only of FliO could improve motility of cells encoding a *fliO* gene deletion, with or without additional bypass mutations in *fliP*.

In *Buchnera* sp. APS a gene fusion of *fliO* and *fliP* exists [41]. This suggests along with the results presented here that FliO and FliP probably also interact in *Salmonella*. This is important because FliP has been demonstrated to be located within the flagellar basal body [4]. FliP is predicted to contain 4 transmembrane loops with the 2^nd^ and 3^rd^ loops connected by a large (approximately 80-residue) periplasmic domain (Figure S2). The nature of the bypass mutations described here in the primary sequence of FliP, appear to be remarkably subtle. The bypass mutation F190L is located in predicted transmembrane loop 3, and was physiologically relevant by itself at improving motility in a *fliO* deletion background. However, the F190L mutation did not improve levels of FliP suggesting that the mutation is a gain-of-function mutation that overcomes the supporting role of FliO through a different mechanism. The FliP(R143H) mutation alone was not sufficient to greatly improve motility, rather it somehow increases the spectrum of bypass mutations permissible for rescuing motility of a *fliO* deletion mutant. Since arginine 143 is located in the periplasm, mutations might occur in other proteins, which interact with this domain. We are presently mapping the additional bypass mutation in strain CB191, which exists together with the *fliP*(*R143H*) bypass mutation. FliP(R143H) and FliP(F190L) do not correspond to residues found at the same position for the *A. aeolicus* FliP orthologue or the *Salmonella* serovar Typhimurium FliP needle paralogues, SsaR and SpaP, which are all found in type III secretion systems without a FliO homologue (Figure S2). So these FliP homologues might consist of amino acid residues that increase their stability, or they are part of secretion systems, which do not require the supporting role(s) that FliO plays in the *Salmonella* system.

The most highly conserved part of the FliO protein is between residues 22 to 95. This is consistent with the results of the truncation analysis, which showed that the periplasmic domain and C-terminal 30 amino acid residues are non-essential. However, mutating residue leucine 91 of the cytoplasmic domain severely disrupted FliO function. Furthermore, overexpression of the cytoplasmic domain of FliO can partially rescue motility of a Δ*fliO* mutant, demonstrating functionality of this domain. Presumably, overexpression of the cytoplasmic domain of FliO overcomes the localization defect of not producing the transmembrane domain, and so overexpression enables the cytoplasmic domain to find its interaction partner(s). However, it is intriguing to know how this domain can function without being anchored to the membrane, since this suggests it might function as bridging domain to maintain protein complex stability, rather than having a catalytic role in protein translocation? Using circular dichroism spectroscopy we showed that the structure of the cytoplasmic domain FliO_43–125_-Ala is a mixture of beta-sheet and random coil. We assume that the FliO cytoplasmic domain becomes structured while interacting with its binding partners similar to binding domains of many other proteins that acquire tertiary structure upon binding to their partners, such as in flagellin/flagellin or tropomodulin/tropomyosin interactions [42], [43].

## Materials and Methods

### Bacterial strains and plasmids

The bacterial strains and plasmids used in this study are listed (Table S3 and Table S4, respectively). Soft-tryptone motility agar, 0.35% (w/v), was used in motility assays [44]. Motility agar plates were maintained at 30°C, and inoculated from colonies from fresh overnight transformations. Ampicillin was used in media at 100 µg ml^−1^ for *Salmonella* strains and 50 µg ml^−1^ for *E. coli* strains. Kanamycin was used at 50 µg ml^−1^, tetracycline at 15 µg ml^−1^, chloramphenicol at 34 µg ml^−1^, and 5-Bromo-4-chloro-3-indoyl phosphate at 40 µg ml^−1^, where applicable. All chemicals were obtained from Sigma-Aldrich or Wako, Japan.

### Genetic engineering procedures

The oligonucleotides used in the strain and plasmid constructions are listed (Table S5 and Table S6, respectively). To construct chromosomal gene deletions and replacements *λ*-Red-based recombination was used employing plasmid pKD46 [45], [46]. To construct chromosomal gene deletions a kanamycin-resistance cassette was obtained from plasmid pKD13 by PCR, flanked with approximately 40-bp ends homologous to the target site. After chromosomal integration the kanamycin resistance cassette was excised using plasmid pCP20 so that the ‘scar’, which remains afterwards within the target gene(s) was encoded as an in-frame short polypeptide for the non-polar deletions, or the ‘scar’ was in the reverse orientation for the Δ*phoN301* allele. The Δ*fliO22252* deletion encodes the first 5 residues, and the final 8 residues of FliO, linked by 27-amino acid residues from an internal ‘scar’ sequence within the *fliO* gene. The Δ(*fliO-fliP*)*22251* deletion encodes the first 5 residues of FliO and the last 5 residues of FliP, joined by 28 residues encoded by a scar sequence.

To construct chromosomal gene replacements a *tetRA* tetracycline-resistance cassette was obtained by PCR from SGSC3718 genomic DNA flanked by approximately 40-bp ends homologous to the target site. After chromosomal integration, it was possible to counter-select against the tetracycline-resistance cassette on medium containing fusaric acid. Then the targeted region of the chromosome was replaced using PCR-amplified DNA containing homologous-ends. To create *fliO*::*phoA* or *fliO*::*gfpuv* chimeric gene fusions in plasmid pTSO17, which carries the *fliO* gene, it was first linearized at the desired point by non-strand displacing PCR. Then either a *phoA* gene PCR product obtained from *E. coli* K-12 MG1655 genomic DNA or a GFPuv gene PCR product obtained from plasmid pGFPuv (Clontech), which was flanked by 15-bp *fliO* homologous ends, was inserted by the homologous recombination-based In-fusion PCR cloning procedure (Clontech). Site-directed mutagenesis was performed using the QuickChange Lightning Site-Directed Mutagenesis Kit (Stratagene).

### Purification of FliO_43–125_-Ala and FliO_43–125_-6xHis

FliO_43–125_-Ala was purified using a C-terminal intein tag encoded by a pTXB1-based expression plasmid, according to manufacturer's instructions (New England Biolabs). BL21 Star containing plasmid pTSO133 was cultivated. The cell pellet from 5-L culture was suspended in 200-ml buffer consisting of 40 mM Tris-HCl (pH 8.0), 1 M NaCl, 2 mM EDTA, and 20% glycerol, and lysed by sonication. Insoluble proteins and cell debris were collected by low-speed centrifugation and suspended in 100-ml buffer containing 40 mM Tris-HCl (pH 8.0), 0.5 M NaCl, and 8 M urea. The suspension was incubated with stirring at room temperature for 3 hours. Insoluble material was removed by centrifugation. After centrifugation, the supernatant was diluted four times with washing buffer consisting of 40 mM Tris-HCl (pH 8.0), 0.5 M NaCl, and 1 M urea, and loaded onto a chitin bead column equilibrated with washing buffer (the column volume was 30-ml). The column was washed with 200-ml of washing buffer, and then with 100-ml of washing buffer containing 0.1 M DTT. After that the column was incubated at room temperature for about 20 hours. FliO_43–125_-Ala was eluted from the chitin column with 50-ml of washing buffer and further purified by gel-filtration on a HiLoad Superdex-75 gel filtration column (GE healthcare) equilibrated with the same buffer. Fractions containing pure FliO_43–125_-Ala were pooled together, and the protein was transferred by dialysis in 20 mM Na/K phosphate (pH 6.2), and 100 mM NaCl. FliO_43–125_-6xHis was purified according to standard procedures, using nickel affinity chromatography under denaturing conditions after expression from plasmid pESO221.

### Immunoblotting

Immunoblotting was performed similar to a previously described method [25]. For immunoblotting of whole cell lysates, colonies of *Salmonella* serovar Typhimurium containing the desired plasmid, from an overnight transformation, were inoculated into 5-ml LB with antibiotic and grown with shaking for 6 hours at 37°C. Cells were harvested by centrifugation at 16,100-*g* for 5 minutes, and the pellets were re-suspended in SDS-PAGE loading buffer (100 mM Tris-HCl, pH 6.8, 2% SDS, 1 mM *β*-mercaptoethanol, 7 M urea, and 0.1% bromophenol blue) and incubated at 95°C for 15 minutes. An equivalent amount of each whole cell lysate was separated by SDS PAGE and proteins were either detected by staining with Coomassie brilliant blue or transferred to PVDF membranes. Immunoblotting was performed using the anti-rabbit WesternBreeze Chromogenic Kit (Invitrogen), according to manufacturer's instructions. The following antibodies and dilutions were used: anti-alkaline phosphatase (Rockland, PA, USA), 1∶20,000 dilution; anti-GFP (Clontech), 1∶5,000 dilution; ANTI-FLAG (Sigma-Aldrich), 1∶5,000 dilution; and polyclonal anti-FliO_43–125_-6xHis antibody, 1∶10,000 dilution.

### Cell fractionation

Cells were separated into the insoluble membrane pellet fraction and soluble supernatant fraction after lysis similar to a previously described method [47]. Briefly, from overnight transformations, colonies of *Salmonella* serovar Typhimurium containing the desired plasmid were inoculated into 50-ml LB with antibiotic and grown with shaking for 7 hours at 37°C. Cells were harvested by centrifugation at 6000-*g* for 10 minutes, and the pellets were re-suspended in 11-ml 50 mM Tris-HCl (pH 8.0), 10 mM MgCl_2_, and 440-µl 25× EDTA-free protease inhibitor cocktail (Roche). Cell pellets were lysed by sonication at 25% power for 5 cycles of one minute each. After incubation at room temperature for 30 minutes unlysed cell debris was removed by low-speed centrifugation at 10,000-*g* for 10 minutes. The insoluble membrane pellet was obtained by high-speed centrifugation at 100,000-*g* for 1 hour and resuspended in 1-ml 50 mM Tris-HCl (pH 8.0), and 10 mM MgCl_2_. One-µl was added to 5-µl SDS-PAGE loading buffer for electrophoresis. The supernatant was further clarified by centrifugation at 100,000-*g* for 1 hour and 10-µl was added to 5-µl SDS-PAGE loading buffer for electrophoresis.

### Observation of GFPuv fluorescence and measurement of alkaline phosphatase activity

Expression of FliO/GFPuv chimeras was detected for strains inoculated onto L-agar plates containing the appropriate antibiotic, after 4 days incubation at 30°C, using a transilluminator set at 365 nm UV. Alkaline phosphatase assays are detailed in Text S1.

### Circular Dichroism (CD) Spectroscopy

CD spectra of FliO_43–125_-Ala was measured using an Aviv model 400 spectropolarimeter (Lakewood, NJ) in 0.1 cm cuvettes at 0°C in 20 mM Na/K phosphate, pH 6.2; 100 mM NaCl. Urea titration ellipticity at 220 nm was measured for samples in 0, 1, 2, 3, 4, 5, 6, 7 and 8 M urea at 10°C.

### Bioinformatics

Protein secondary structure prediction was performed using Jpred 3 [48]. Programs used to align protein sequences, and programs used to predict membrane protein-membrane topology are detailed in Text S1.

## Supporting Information

Figure S1 Alignment and predicted membrane topologies of FliO proteins from *Gammaproteobacteria*. SALTY  =  *Salmonella enterica* serovar Typhimurium; ECOLI  =  *Escherichia coli* K-12; ERWTA  =  *Erwinia tasmaniensis*; YEREN  =  *Yersinia enterocolitica*; XANCA  =  *Xanthomonas campestris*; VIBCH  =  *Vibrio cholerae*; and PSEAE  =  *Pseudomonas aeruginosa*. We have shown for *Salmonella* serovar Typhimurium FliO that the underlined residues in bold type could be truncated from the N-terminus or the C-terminus, and FliO still remained partially functional. We also identified residue leucine 91 (black bold-type) is important for the function of full-length *Salmonella* serovar Typhimurium FliO. The percentage score of the FliO homologues with *Salmonella* serovar Typhimurium FliO is indicated.
(0.03 MB PDF)

Figure S2 Alignment and predicted membrane topologies of FliP, and FliP homologues. *Salmonella enterica* serovar Typhimurium FliP, the *Aquifex aeolicus* FliP orthologue, and the *Salmonella* serovar Typhimurium FliP paralogues, SpaP and SsaR were aligned. SALTY  =  *Salmonella* serovar Typhimurium, and AQUAE  = *A. aeolicus*. *A. aeolicus* FliP, and SpaP and SsaR are found in Type III secretion systems without a FliO homologue. In this study, it was shown that bypass mutations in *Salmonella* serovar Typhimurium FliP corresponding to R143H and F190L, could partially rescue motility of a *fliO* deletion mutant. Residues aligned with arginine 143 and phenylalanine 190 of *Salmonella* serovar Typhimurium FliP are indicated in dark red bold type. The percentage score of the homologues with *Salmonella* serovar Typhimurium FliP is indicated.
(0.03 MB PDF)

Table S1 Prediction of FliO transmembrane topology.
(0.05 MB DOC)

Table S2 Alkaline phosphatase activity of *Salmonella enterica* serovar Typhimurium expressing chimeric fusions of alkaline phosphatase within FliO.
(0.03 MB DOC)

Table S3 Strains used in this study.
(0.06 MB DOC)

Table S4 Plasmids used in this study.
(0.06 MB DOC)

Table S5 Oligonucleotides used in strain constructions.
(0.06 MB DOC)

Table S6 Oligonucleotides used in plasmid constructions.
(0.12 MB DOC)

Text S1 Supplementary materials and methods.
(0.03 MB DOC)

## References

[1] Blocker A, Komoriya K, Aizawa S-I (2003). Type III secretion systems and bacterial flagella: Insights into their function from structural similarities.. Proc Natl Acad Sci U S A.

[2] Macnab RM (2003). How bacteria assemble flagella.. Annu Rev Microbiol.

[3] Macnab RM (2004). Type III flagellar protein export and flagellar assembly.. Biochim Biophys Acta.

[4] Fan F, Ohnishi K, Francis NR, Macnab RM (1997). The FliP and FliR proteins of *Salmonella typhimurium*, putative components of the type III flagellar export apparatus, are located in the flagellar basal body.. Mol Microbiol.

[5] Kihara M, Minamino T, Yamaguchi S, Macnab RM (2001). Intergenic suppression between the flagellar MS ring protein FliF of *Salmonella* and FlhA, a membrane component of its export apparatus.. J Bacteriol.

[6] Van Arnam JS, McMurry JL, Kihara M, Macnab RM (2004). Analysis of an engineered *Salmonella* flagellar fusion protein, FliR-FlhB.. J Bacteriol.

[7] Minamino T, Macnab RM (1999). Components of the *Salmonella* flagellar export apparatus and classification of export substrates.. J Bacteriol.

[8] Chevance FFV, Hughes KT (2008). Coordinating assembly of a bacterial macromolecular machine.. Nat Rev Microbiol.

[9] Minamino T, Imada K, Namba K (2008). Mechanisms of type III protein export for bacterial flagellar assembly.. Mol BioSyst.

[10] Minamino T, Namba K (2008). Distinct roles of the FliI ATPase and proton motive force in bacterial flagellar protein export.. Nature.

[11] Paul K, Erhardt M, Hirano T, Blair DF, Hughes KT (2008). Energy source of flagellar type III secretion.. Nature.

[12] Saijo-Hamano Y, Imada K, Minamino T, Kihara M, Shimada M (2010). Structure of the cytoplasmic domain of FlhA and implication for flagellar type III protein export.. Mol Microbiol.

[13] Moore SA, Jia Y (2010). Structure of the cytoplasmic domain of the flagellar secretion apparatus component FlhA from *Helicobacter pylori*.. J Biol Chem.

[14] Worrall LJ, Vuckovic M, Strynadka NC (2010). Crystal structure of the C-terminal domain of the *Salmonella* type III secretion system export apparatus protein InvA.. Protein Sci.

[15] Zarivach R, Deng W, Vuckovic M, Felise HB, Nguyen HV (2008). Structural analysis of the essential self-cleaving type III secretion proteins EscU and SpaS.. Nature.

[16] Deane JE, Graham SC, Mitchell EP, Flot D, Johnson S (2008). Crystal structure of Spa40, the specificity switch for the *Shigella flexneri* type III secretion system.. Mol Microbiol.

[17] Lountos GT, Austin BP, Nallamsetty S, Waugh DS (2009). Atomic resolution structure of the cytoplasmic domain of *Yersinia pestis* YscU, a regulatory switch involved in type III secretion.. Prot Sci.

[18] Minamino T, Macnab RM (2000). Interactions among components of the *Salmonella* flagellar export apparatus and its substrates.. Mol Microbiol.

[19] Fraser GM, González-Pedrajo B, Tame JRH, Macnab RM (2003). Interactions of FliJ with the *Salmonella* type III flagellar export apparatus.. J Bacteriol.

[20] McMurry JL, Van Arnam JS, Kihara M, Macnab RM (2004). Analysis of the cytoplasmic domains of *Salmonella* FlhA and interactions with components of the flagellar export machinery.. J Bacteriol.

[21] Saijo-Hamano Y, Minamino T, Macnab RM, Namba K (2004). Structural and functional analysis of the C-terminal cytoplasmic domain of FlhA, an integral membrane component of the type III flagellar protein export apparatus in *Salmonella*.. J Mol Biol.

[22] Minamino T, Shimada M, Okabe M, Saijo-Hamano Y, Imada K (2010). Role of the C-terminal cytoplasmic domain of FlhA in bacterial flagellar type III protein export.. J Bacteriol.

[23] Minamino T, González-Pedrajo B, Yamaguchi K, Aizawa S-I, Macnab RM (1999). FliK, the protein responsible for flagellar hook length control in *Salmonella*, is exported during hook assembly.. Mol Microbiol.

[24] Minamino T, Macnab RM (2000). Domain structure of *Salmonella* FlhB, a flagellar export component responsible for substrate specificity switching.. J Bacteriol.

[25] Fraser GM, Hirano T, Ferris HU, Devgan LL, Kihara M (2003). Substrate specificity of type III flagellar protein export in *Salmonella* is controlled by subdomain interactions in FlhB.. Mol Microbiol.

[26] Ferris HU, Furukawa Y, Minamino T, Kroetz MB, Kihara M (2005). FlhB regulates ordered export of flagellar components via autocleavage mechanism.. J Biol Chem.

[27] Minamino T, Ferris HU, Moriya N, Kihara M, Namba K (2006). Two parts of the T3S4 domain of the hook-length control protein FliK are essential for the substrate specificity switching of the flagellar type III export apparatus.. J Mol Biol.

[28] Moriya N, Minamino T, Hughes KT, Macnab RM, Namba K (2006). The type III flagellar export specificity switch is dependent on FliK ruler and a molecular clock.. J Mol Biol.

[29] Erhardt M, Hirano T, Su Y, Paul K, Wee DH (2010). The role of the FliK molecular ruler in hook-length control in *Salmonella enterica*.. Mol Micro.

[30] Malakooti J, Komeda Y, Matsumura P (1989). DNA sequence analysis, gene product identification, and localization of flagellar motor components of *Escherichia coli*.. J Bacteriol.

[31] Malakooti J, Ely B, Matsumura P (1994). Molecular characterization, nucleotide sequence, and expression of the *fliO*, *fliP*, *fliQ*, and *fliR* genes of *Escherichia coli*.. J Bacteriol.

[32] Ohnishi K, Fan F, Schoenhals GJ, Kihara M, Macnab RM (1997). The FliO, FliP, FliQ, and FliR proteins of *Salmonella typhimurium*: putative components for flagellar assembly.. J Bacteriol.

[33] Schoenhals GJ, Kihara M, Macnab RM (1998). Translation of the flagellar gene *fliO* of *Salmonella typhimurium* from putative tandem starts.. J Bacteriol.

[34] Liu R, Ochman H (2007). Stepwise formation of the bacterial flagellar system.. Proc Natl Acad Sci U S A.

[35] Pallen MJ, Penn CW, Chaudhuri RR (2005). Bacterial flagellar diversity in the post-genomic era.. Trends Microbiol.

[36] Manoil C, Beckwith J (1986). A genetic approach to analyzing membrane protein topology.. Science.

[37] Ehrmann M, Boyd D, Beckwith J (1990). Genetic analysis of membrane protein topology by a sandwich gene fusion approach.. Proc Natl Acad Sci U S A.

[38] Boyd D, Traxler B, Beckwith J (1993). Analysis of the topology of a membrane protein by using a minimum number of alkaline phosphatase fusions.. J Bacteriol.

[39] Feilmeier BJ, Iseminger G, Schroeder D, Webber H, Phillips GJ (2000). Green fluorescent protein functions as a reporter for protein localization in *Escherichia coli*.. J Bacteriol.

[40] Richardson JS, Richardson DC (2002). Natural *β*-sheet proteins use negative design to avoid edge-to-edge aggregation.. Proc Natl Acad Sci U S A.

[41] Shigenobu S, Watanabe H, Hattori M, Sakaki Y, Ishikawa H (2000). Genome sequence of the endocellular bacterial symbiont of aphids *Buchnera* sp. APS.. Nature.

[42] Kostyukova AS, Pyatibratov MG, Filimonov VV, Federov OV (1988). Flagellin parts acquiring a regular structure during polymerization are disposed on the molecule ends.. FEBS Lett.

[43] Kostyukova AS, Tiktopulo EI, Maéda Y (2001). Folding properties of functional domains of tropomodulin.. Biophys J.

[44] Toker AS, Kihara M, Macnab RM (1996). Deletion analysis of the FliM flagellar switch protein of *Salmonella typhimurium*.. J Bacteriol.

[45] Datsenko KA, Wanner BL (2000). One-step inactivation of chromosomal genes in *Escherichia coli* K-12 using PCR products.. Proc Natl Acad Sci USA.

[46] Karlinsey JE (2007). *λ*-Red genetic engineering in *Salmonella enterica* serovar Typhimurium.. Methods Enzymol.

[47] Ethier J, Boyd JM (2000). Topological analysis and role of the transmembrane domain in polar targeting of PilS, a *Pseudomonas aeruginosa* sensor kinase.. Mol Microbiol.

[48] Cole C, Barber JD, Barton GJ (2008). The Jpred 3 secondary structure prediction server.. Nucleic Acids Res.

