# Supplementary material for: FliO Regulation of FliP in the Formation of the Salmonella enterica Flagellum
Source: PLoS Genet. 2010 Sep 30;6(9):e1001143. doi: 10.1371/journal.pgen.1001143 (PMC2947984; doi:10.1371/journal.pgen.1001143)
Supplement: Figure S2 — Alignment and predicted membrane topologies of FliP, and FliP homologues. Salmonella enterica serovar Typhimurium FliP, the Aquifex aeolicus FliP orthologue, and the Salmonella serovar Typhimurium FliP paralogues, SpaP and SsaR were aligned. SALTY = Salmonella serovar Typhimurium, and AQUAE = A. aeolicus. A. aeolicus FliP, and SpaP and SsaR are found in Type III secretion systems without a FliO homologue. In this study, it was shown that bypass mutations in Salmonella serovar Typhimurium FliP corresponding to R143H and F190L, could partially rescue motility of a fliO deletion mutant. Residues aligned with arginine 143 and phenylalanine 190 of Salmonella serovar Typhimurium FliP are indicated in dark red bold type. The percentage score of the homologues with Salmonella serovar Typhimurium FliP is indicated. (0.03 MB PDF) [file pgen.1001143.s002.pdf]

```
                    SIGNAL PEPTIDE          PERIPLASM           MEMBRANE
SALTY_FliP      MRR-LLFLSLAGLWLFSPAAAAQLPGLISQPLAGGGQSWSLSVQTLVFITSLTFLPAILL 59
AQUAE_FliP      MRKALTILILSAGFVFS-----QIPPI---ELKVGEGNLVDSIRLLIFLTILSLVPSILI 52
SpaP            --------------------------------------MGNDISLIALLAFSTLLPFIIA 22
SsaR            ---------------------MSLPDS--------------PLQLIGILFLLSILPLIIV 25
                                                       * *

                                CYTOPLASM       MEMBRANE
SALTY_FliP      MMTSFTRIIIVFGLLRNALGTPSAPPNQVLLGLALFLTFFIMSPVIDKIYVDAYQP--FS 117
AQUAE_FliP      MFTSFTRLVVVLSLLRQAIGTPQAPPNQVIIALSLFLTFFIMKPTIDKINSEALQP--YI 110
SpaP            SGTCFVKFSIVFVMVRNALGLQQIPSNMTLNGVALLLSMFVMWPIMHDAYVYFEDEDVTF 82
SsaR            MGTSFLKLAVVFSILRNALGIQQVPPNIALYGLALVLSLFIMGPTLLAVK-ERWHPVQVA 84
                  * *     *    * * *    * *       * *  * * *

                                           PERIPLASM
SALTY_FliP      EQKISMQEALDKGAQPLRAFMLRQTREADLALFARL-AN----SGP------LQGPEAVP 166
AQUAE_FliP      REEISDEEFFKRVEFYAKDFMLKHTRKETLEAFLSI-AK--IPKDS------VKEPHEIP 161
SpaP            NDISSLSKHVDEGLDGYRDYLIKYSDRELVQFFENAQLKRQYGEETETVKRDKDEIEKPS 142
SsaR            GAPFWTSEWDSKALAPYRQFLQKNSEEKEANYFRNL-IKRTWPEDI------KRKIKPDS 137
                                                *

                                           MEMBRANE          CYTOPLASM
SALTY_FliP      MRILLPAYVTSELKTAFQIGFTIFIPFLIIDLVIASVLMALGMMMVPPATIALPFKLMLF 226
AQUAE_FliP      LRVVIPAFMVSELKTAFEIVFLLYIPFLIVDLVVASILISMGIIMIPPQLISLPFKIMLF 221
SpaP            IFALLPAYALSEIKSAFKIGFYLYLPFVVVDLVVSSVLLALGMMMMSPVTISTPIKLVLF 202
SsaR            LLILIPAFTVSQLTQAFRIGLLIYLPFLAIDLLISNILLAMGMMMVSPMTISLPFKLLIF 197
                     **   *    ** *      **   **     *   *  *  *  *  * *   *

                    MEMBRANE    PERIPLASM
SALTY_FliP      VLVDGWQLLMGSLAQSFYS--- 245
AQUAE_FliP      VLANGWELVVLSLVRSYQ---- 239     43 %
SpaP            VALDGWTLLSKGLILQYMDIAT 224     32 %
SsaR            LLAGGWDLTLAQLVQSFS---- 215     37 %
                    ** *    *
```
