# Supplementary material for: FliO Regulation of FliP in the Formation of the Salmonella enterica Flagellum
Source: PLoS Genet. 2010 Sep 30;6(9):e1001143. doi: 10.1371/journal.pgen.1001143 (PMC2947984; doi:10.1371/journal.pgen.1001143)
Supplement: Table S1 — Prediction of FliO transmembrane topology. (0.05 MB DOC) [file pgen.1001143.s003.doc]

Table S1. Prediction of FliO transmembrane topology

| Program | Number of transmembrane segments | Position in FliO  (125 amino acid residues) | Orientation^a^ |
| --- | --- | --- | --- |
| Phobius | 1 | Residues 20-40 | N = periplasm |
|  |  |  | C = cytoplasm |
| TMpred | 1 | Residues 19-40 | N = periplasm |
|  |  |  | C = cytoplasm |
| MEMSAT3 | 1 | Residues 18-42 | N = periplasm |
|  |  |  | C = cytoplasm |
| ConPred II | 1 | Residues 20-40 | N = periplasm |
|  |  |  | C = cytoplasm |
| TMHMM 2.0 | 1 | Residues 18-40 | N = periplasm |
|  |  |  | C = cytoplasm |
| HMMTOP 2.0 | 1 | Residues 22-40 | N = periplasm |
|  |  |  | C = cytoplasm |
| PHDhtm | 1 | Residues 23-40 | N = periplasm |
|  |  |  | C = cytoplasm |
| TMAP | 1 | Residues 17-43 | N = cytoplasm |
|  |  |  | C = periplasm |
| TopPred II^b^ | 1 | Residues 20-40 | N = cytoplasm |
|  |  |  | C = periplasm |
| TopPred II^c^ | 2 | Residues 21-41 and 67-87 | N = periplasm |
|  |  |  | C = periplasm |
| DAS | 2 | Residues 22-39 and 82-83 | Not Determined |

^a^ N, N-terminus. C, C-terminus.

^b^ Using hydrophobicity file: GES-scale

^c^ Using hydrophobicity file: KD-scale
