# Supplementary material for: FliO Regulation of FliP in the Formation of the Salmonella enterica Flagellum
Source: PLoS Genet. 2010 Sep 30;6(9):e1001143. doi: 10.1371/journal.pgen.1001143 (PMC2947984; doi:10.1371/journal.pgen.1001143)
Supplement: Table S2 — Alkaline phosphatase activity of Salmonella enterica serovar Typhimurium expressing chimeric fusions of alkaline phosphatase within FliO. (0.03 MB DOC) [file pgen.1001143.s004.doc]

Table S2. Alkaline phosphatase activity of Salmonella enterica serovar Typhimurium expressing chimeric fusions of alkaline phosphatase within FliO

| Strain | Fusion | Chromosomal expression^a^, ^b^ | Chromosomal and plasmid expression^a^, ^c^ |
| --- | --- | --- | --- |
| CB288 | FliO_1-6_::PhoA_22-471_::FliO_7-125_ | 0.9 | 20.4 |
| CB290 | FliO_1-100_::PhoA_22-471_::FliO_101-125_ | 0.0 | 0.2 |
| CB291 | FliO_1-115_::PhoA_22-471_::FliO_116-125_ | 0.1 | 0.3 |

^a^ All assays were performed in triplicate; standard deviations were less than 10%; alkaline phosphatase activities of strains CB288, CB290, and CB291 (all ΔphoN301) expressing each fusion were normalized against activity for strain CB284 (fliO^+^ ΔphoN301) which does not express phosphatase.

^b^ For chromosomal expression of the chimeric fusions, the gene for the mature form of alkaline phosphatase, phoA, was genetically engineered into the fliO gene, at the desired location, at the native site of fliO on the chromosome.

^c^ For chromosomal and plasmid expression, the same fusion was expressed from plasmid pTrc99A-FF4 (without IPTG induction) and the chromosome.
