# Supplementary material for: FliO Regulation of FliP in the Formation of the Salmonella enterica Flagellum
Source: PLoS Genet. 2010 Sep 30;6(9):e1001143. doi: 10.1371/journal.pgen.1001143 (PMC2947984; doi:10.1371/journal.pgen.1001143)
Supplement: Table S3 — Strains used in this study. (0.06 MB DOC) [file pgen.1001143.s005.doc]

Table S3. Strains used in this study

| Strain | Genotype or relevant characteristic | Source |
| --- | --- | --- |
| Escherichia coli | | |
| DH5α; XL10-Gold; Fusion-Blue | Recipients for cloning experiments | Invitrogen; Stratagene; Clontech |
| BL21 Star | For overproduction of proteins from pET-based plasmids | Invitrogen |
| MG1655 | K-12, wild-type; source of the phoA gene | NIG ME7986^a^; 1 |
| BW25113 | Plasmid pKD46; Amp^R^; temperature-sensitive ori 30^o^C | CGSC7739^b^ |
| BW25141 | Plasmid pKD13; Amp^R^ and Km^R^ | CGSC7633^b^ |
| BT340 | DH5α with plasmid pCP20; Amp^R^ and Cam^R^; temperature-sensitive ori 30^o^C | CGSC7629^b^ |
| Salmonella enterica serovar Typhimurium | | |
| JR501 | R^-^m^+^ for converting plasmids to Salmonella compatibility | 2 |
| SJW1103 | Wild-type for motility and chemotaxis | 3 |
| TT13206 | LT7; phoN51::Tn10-11(Tet^R^); source of tetRA (Tet^R^) genes | SGSC3718^c^; 4 |
| CB173 | ∆(fliO-fliP)22251::km | This study |
| CB176 | ∆fliO22252::km | This study |
| CB182 | ∆fliO22253::tetRA(Tet^R^) | This study |
| CB184 | ∆(fliO-fliP)22251 | This study |
| CB186 | ∆fliO22252 | This study |
| CB191 | ∆fliO22252 fliP22254(R143H) (base change G428A of fliP); motile pseudorevertant strain derived from CB186 incubated in motility agar | This study |
| CB227 | ∆fliO22252 fliP22255(F190L) (base change T568C of fliP); motile pseudorevertant strain derived from CB186 incubated in motility agar | This study |
| CB269 | ∆fliO22253::tetRA(Tet^R^) ∆phoN301::km | This study |
| CB271 | ∆fliO22253::tetRA(Tet^R^) ∆phoN301 | This study |
| CB274 | ∆(fliO-fliP)22256::tetRA(Tet^R^) | This study |
| CB281 | ∆fliO22252 fliP22254(R143H) | This study |
| CB282 | ∆fliO22252 fliP22255(F190L) | This study |
| CB284 | fliO^+^ ∆phoN301 | This study |
| CB288 | Φ(fliO[1-6]-phoA_E. coli_[22-471]-fliO[7-125])22257(Hyb) ∆phoN301 | This study |
| CB290 | Φ(fliO[1-100]-phoA_E. coli_[22-471]-fliO[101-125])22258(Hyb) ∆phoN301 | This study |
| CB291 | Φ(fliO[1-115]-phoA_E. coli_[22-471]-fliO[116-125])22259(Hyb) ∆phoN301 | This study |
| CB308 | fliO22262(L91A) | This study |
| CB309 | fliO22263(∆91) | This study |
| CB310 | fliP22254(R143H) | This study |
| CB311 | fliP22255(F190L) | This study |

^a^ NIG, National Institute of Genetics, National BioResource Project (NIG, Japan): E. coli., Shizuoka, Japan.

^b^ CGSC, Escherichia coli Genetic Stock Center, Yale University, New Haven, Connecticut, USA.

^c^ SGSC, Salmonella Genetic Stock Centre, University of Calgary, Calgary, Alberta, Canada.

1. Blattner FR, Plunkett III G, Bloch CA, Perna NT, Burland V, et al. (1997) The complete genome sequence of Escherichia coli K-12. Science 277: 1453-1462.

2. Ryu J-I, Hartin RJ (1990) Quick transformation in Salmonella typhimurium LT2. BioTechniques 8: 43-45.

3. Yamaguchi S, Fujita H, Sugata K, Taira T, Iino T (1984) Genetic analysis of H2, the structural gene for phase-2 flagellin in Salmonella. J Gen Microbiol 130: 255-265.

4. Jiang W, Metcalf WW, Lee K-S, Wanner BL (1995) Molecular cloning, mapping, and regulation of Pho regulon genes for phosphonate breakdown by the phosphonatase pathway of Salmonella typhimurium LT2. J Bacteriol 177: 6411-6421.
