# Supplementary material for: FliO Regulation of FliP in the Formation of the Salmonella enterica Flagellum
Source: PLoS Genet. 2010 Sep 30;6(9):e1001143. doi: 10.1371/journal.pgen.1001143 (PMC2947984; doi:10.1371/journal.pgen.1001143)
Supplement: Table S4 — Plasmids used in this study. (0.06 MB DOC) [file pgen.1001143.s006.doc]

Table S4. Plasmids used in this study

| Plasmid | Description | Source |
| --- | --- | --- |
| pTXB1 | pT7 expression vector, C-terminal intein tag; Amp^R^ | New England Biolabs |
| pET-22b(+) | pT7 expression vector, optional C-terminal His-tag; Amp^R^ | Novagen |
| pGFPuv | Plasmid containing the GFPuv gene; Amp^R^ | Clontech |
| pKD46 | *λ*-Red genetic engineering plasmid; Amp^R^; temperature-sensitive ori 30^o^C | CGSC^a^; 1 |
| pKD13 | PCR template for isolation of an FRT-flanked kanamycin resistance cassette for *λ*-Red genetic engineering; Amp^R^ and Km^R^ | CGSC^a^; 1 |
| pCP20 | For Flp-catalyzed excision of the kanamycin-resistance determinant isolated from pKD13 after *λ*-Red genetic engineering; Amp^R^ and Cam^R^; temperature-sensitive ori 30^o^C. | CGSC^a^; 1, 2 |
| pTrc99A-FF4 | Modified pTrc99A expression vector; Amp^R^ | 3 |
| pTSO17 | pTrc99A-FF4 carrying full-length fliO | This study |
| pTSO18 | pTrc99A-FF4 carrying fliO codons 22 to 125 (N-terminal methionine residue encoded at position 22 instead of a natural valine residue) | This study |
| pTSO19 | pTrc99A-FF4 carrying fliO codons 43 to 125 | This study |
| pTSO20 | pTrc99A-FF4 carrying full-length fliO with artificial -TAATAG-3` tandem stop codons at the -3` end of the fliO gene | This study |
| pTSO22 | pTrc99A-FF4 carrying fliO codons 43 to 125 with artificial -TAATAG-3` tandem stop codons at the -3’ end of the fliO gene | This study |
| pTSO25 | pTrc99A-FF4 carrying fliO codons 1 to 65 | This study |
| pTSO26 | pTrc99A-FF4 carrying fliO codons 1 to 75 | This study |
| pTSO27 | pTrc99A-FF4 carrying fliO codons 1 to 85 | This study |
| pTSO28 | pTrc99A-FF4 carrying fliO codons 1 to 95 | This study |
| pTSO29 | pTrc99A-FF4 carrying fliO codons 1 to 105 | This study |
| pTSO30 | pTrc99A-FF4 carrying fliO codons 1 to 115 | This study |
| pTSO133 | pTXB1 carrying fliO codons 43 to 125 and a -3` alanine codon followed by codons encoding an intein fusion | This study |
| pTSO162 | pTrc99A-FF4 carrying fliO(∆91) | This study |
| pTSO193 | pTrc99A-FF4 carrying a fliO::phoA chimeric fusion with E. coli phoA codons 22 to 471, sandwiched in-between codons 6 and 7 of Salmonella serovar Typhimurium fliO | This study |
| pTSO195 | pTrc99A-FF4 carrying a fliO::phoA chimeric fusion with E. coli phoA codons 22 to 471, sandwiched in-between codons 100 and 101 of Salmonella serovar Typhimurium fliO | This study |
| pTSO196 | pTrc99A-FF4 carrying a fliO::phoA chimeric fusion with E. coli phoA codons 22 to 471, sandwiched in-between codons 115 and 116 of Salmonella serovar Typhimurium fliO | This study |
| pTSO198 | pTrc99A-FF4 carrying a fliO::gfpuv chimeric fusion with the GFPuv gene, sandwiched in-between codons 6 and 7 of Salmonella serovar Typhimurium fliO | This study |
| pTSO201 | pTrc99A-FF4 carrying a fliO::gfpuv chimeric fusion with the GFPuv gene, sandwiched in-between codons 115 and 116 of Salmonella serovar Typhimurium fliO | This study |
| pTSOP210 | pTrc99A-FF4 carrying fliO and fliP | This study |
| pTSP211 | pTrc99A-FF4 carrying fliP | This study |
| pTSO220 | pTrc99A-FF4 carrying fliO codons 43 to 125 with six -3` histidine codons | This study |
| pESO221 | pET-22b(+) carrying fliO codons 43 to 125 with six -3` histidine codons | This study |
| pTSO239 | pTrc99A-FF4 carrying fliO(L91A) | This study |
| pTSOP259 | pTrc99A-FF4 carrying fliO and fliP(R143H) | This study |
| pTSOP260 | pTrc99A-FF4 carrying fliO and fliP(F190L) | This study |
| pTSP324 | pTrc99A-FF4 carrying fliP, with codons for a FLAG-tag (N-DYKDDDDK-C) inserted between codons 22 and 23 of the fliP gene | This study |
| pTSPO325 | pTrc99A-FF4 carrying fliP, with codons for a FLAG-tag (N-DYKDDDDK-C) inserted between codons 22 and 23 of the fliP gene, and also carrying downstream full-length fliO | This study |
| pTSPO326 | pTrc99A-FF4 carrying fliP, with codons for a FLAG-tag (N-DYKDDDDK-C) inserted between codons 22 and 23 of the fliP gene, and also carrying downstream fliO codons 43 to 125 | This study |

^a^ CGSC, Escherichia coli Genetic Stock Center, Yale University, New Haven, Connecticut, USA.

1. Datsenko KA, Wanner BL (2000) One-step inactivation of chromosomal genes in Escherichia coli K-12 using PCR products. Proc Natl Acad Sci U S A 97: 6640-6645.

2. Cherepanov PP, Wackernagel W (1995) Gene disruption in Escherichia coli: Tc^R^ and Km^R^ cassettes with the option of Flp-catalyzed excision of the antibiotic-resistance determinant. Gene 158: 9-14.

3. Ohnishi K, Fan F, Schoenhals GJ, Kihara M, Macnab RM (1997) The FliO, FliP, FliQ, and FliR proteins of Salmonella typhimurium: putative components for flagellar assembly. J Bacteriol 179: 6092-6099.
