# Supplementary material for: FliO Regulation of FliP in the Formation of the Salmonella enterica Flagellum
Source: PLoS Genet. 2010 Sep 30;6(9):e1001143. doi: 10.1371/journal.pgen.1001143 (PMC2947984; doi:10.1371/journal.pgen.1001143)
Supplement: Table S5 — Oligonucleotides used in strain constructions. (0.06 MB DOC) [file pgen.1001143.s007.doc]

Table S5. Oligonucleotides used in strain constructions

| Primer | Name | Use | Pair | Template | Strain | Sequence (5` to 3`) |
| --- | --- | --- | --- | --- | --- | --- |
| 195 | 5`-ΔfliO::km | Forward PCR primer for amplifying kanamycin-resistance cassettes to replace the fliO gene with the ΔfliO22252::km allele, or the fliO and fliP genes with the Δ(fliO-fliP)22251::km allele | 196 or 215 | pKD13 | CB173; CB176 | ccgagcgtatgcgtcgtttgagtcgttaatgatgaagacagaaattccggggatccgtcgacc |
| 196 | 3`-ΔfliOP::km | Reverse PCR primer for amplifying a kanamycin-resistance cassette to replace the fliO and fliP genes with the Δ(fliO-fliP)22251::km allele | 195 | pKD13 | CB173 | gattcaggagtcattttgcgcctctaactgtaaaagctttggggtgtaggctggagctgcttc |
| 215 | 3`-ΔfliO::km | Reverse PCR primer for amplifying a kanamycin-resistance cassette to replace the fliO gene with the ΔfliO22252::km allele | 195 | pKD13 | CB176 | gaataacaaacggcgcatcaggatctcccggaacgcttgagtagtgtaggctggagctgcttc |
| 216 | 5`-ΔfliO::tetRA | Forward PCR primer for amplifying tetracycline-resistance cassettes to replace the fliO gene with the ΔfliO22253::tetRA allele or the fliO and fliP genes with the Δ(fliO-fliP)22256::tetRA allele | 217 or 329 | TT13206 genomic DNA | CB182; CB274 | gtatgcgtcgtttgagtcgttaatgatgaagacagaagccttaagacccactttcacatt |
| 217 | 3`-ΔfliO::tetRA | Reverse PCR primer for amplifying a tetracycline-resistance cassette to replace the fliO gene with the ΔfliO22253::tetRA allele | 216 | TT13206 genomic DNA | CB182 | cggcgcatcaggatctcccggaacgcttgagtaagctcttctaagcacttgtctcctg |
| 245 | 3`-fliOP-245 | Reverse PCR primer for amplifying the fliO gene or fliO::phoA fusions for replacing the ΔfliO22253::tetRA allele | 252 or 330 | pTSO17:  pTSO193; pTSO195; pTSO239; pTSO162 | CB284; CB288; CB290; CB308; CB309 | caaacggcgcatcaggatctc |
| 246 | 3`-fliOP-246 | Reverse PCR primer for amplifying a fliO::phoA fusion for replacing the ΔfliO22253::tetRA allele | 252 | pTSO196 | CB291 | acaaacggcgcatcaggatctc |
| 252 | 5`-fliO-252 | Forward PCR primer for amplifying the fliO gene or fliO::phoA fusions for replacing the ΔfliO22253::tetRA allele | 245, 246, or 340 | pTSO17; pTSO195; pTSO196; pTSO239; pTSO162; pTSOP259; pTSOP260 | CB284; CB290; CB291; CB308; CB309; CB310; CB311 | cgagcgtatgcgtcgtttgagtcgttaatgatgaagacagaagccacgg |
| 257 | 5`-ΔphoN::km | Forward PCR primer for amplifying a kanamycin-resistance cassette to replace the phoN gene with the ΔphoN301::km allele | 258 | pKD13 | CB269 | attattgcctgatccggagtgagtctttatgaaaagtcgtgtgtaggctggagctgcttc |
| 258 | 3`-ΔphoN::km | Reverse PCR primer for amplifying a kanamycin-resistance cassette to replace the phoN gene with the ΔphoN301::km allele | 257 | pKD13 | CB269 | ccagtttgcgggaagactttcaccttcagtaattaagtttattccggggatccgtcgacc |
| 329 | 3`-ΔfliOP::tetRA | Reverse PCR primer for amplifying a tetracycline-resistance cassette to replace the fliO and fliP genes with the Δ(fliO-fliP)22256::tetRA allele | 216 | TT13206 genomic DNA | CB274 | caggagtcattttgcgcctctaactgtaaaagctttgggcctaagcacttgtctcctg |
| 330 | 5`-fliO::phoA | Forward PCR primer for amplifying a fliO::phoA fusion for replacing the ΔfliO22253::tetRA allele | 245 | pTSO193 | CB288 | cgagcgtatgcgtcgtttgagtcgttaatgatgaagacagaagcccggac |
| 338 | 5`-fliN(384-405) | Forward PCR primer for amplifying the fliO and fliP genes to replace the Δ(fliO-fliP)22256::tetRA allele | 339 | CB191; CB227  genomic DNA | CB281; CB282 | atccgagcgtatgcgtcgtttg |
| 339 | 3`-fliQ(-10-14) | Reverse PCR primer for amplifying the fliO and fliP genes to replace the Δ(fliO-fliP)22256::tetRA allele | 338 | CB191; CB227 genomic DNA | CB281; CB282 | gattcaggagtcattttgcgcctc |
| 340 | 3`-fliPQ(717-14) | Reverse PCR primer for amplifying the fliO and fliP genes to replace the Δ(fliO-fliP)22256::tetRA allele | 252 | pTSOP259; pTSOP260 | CB310; CB311 | gattcaggagtcattttgcgcctctaactgtaaaagctttgggcc |
