# Supplementary material for: FliO Regulation of FliP in the Formation of the Salmonella enterica Flagellum
Source: PLoS Genet. 2010 Sep 30;6(9):e1001143. doi: 10.1371/journal.pgen.1001143 (PMC2947984; doi:10.1371/journal.pgen.1001143)
Supplement: Table S6 — Oligonucleotides used in plasmid constructions. (0.12 MB DOC) [file pgen.1001143.s008.doc]

Table S6. Oligonucleotides used in plasmid constructions

| Primer | Name | Use | Pair | Template | Plasmid | Sequence (5` to 3`) |
| --- | --- | --- | --- | --- | --- | --- |
| 1 | 5`-fliO | Forward PCR primer for amplifying the the fliO gene | 18, or 21 to 27; 166 | SJW1103 genomic DNA | pTSO17; pTSO20; pTSO25 to pTSO30; pTSOP210 | gtattcggaaattccatatgatgaagacagaagccacgg |
| 2 | 5`-fliO22 | Forward PCR primer for amplifying the fliO gene from codon 22 | 18 | SJW1103 genomic DNA | pTSO18 | gtattcggaaattccatatgagcggcgcattaattgg |
| 3 | 5`-fliO43 | Forward PCR primer for amplifying the fliO gene from codon 43 | 18, 27 | SJW1103 genomic DNA | pTSO19, pTSO22 | gtattcggaaattccatatgggattcgcgccaaaagg |
| 18 | 3`-fliO125 | Reverse PCR primer for amplifying the fliO gene | 1, 2, or 3 | SJW1103 genomic DNA | pTSO17, pTSO18, pTSO19 | gtcattaaggatccggaataacaaacggcgcatcagg |
| 21 | 3`-fliO65 | Reverse PCR primer for amplifying the fliO gene until codon 65 | 1 | SJW1103 genomic DNA | pTSO25 | gtcattaaggatcctattacgggccaagcgaggcgctg |
| 22 | 3`-fliO75 | Reverse PCR primer for amplifying the fliO gene until codon 75 | 1 | SJW1103 genomic DNA | pTSO26 | gtcattaaggatcctattattcgacctcgacaatcaccac |
| 23 | 3`-fliO85 | Reverse PCR primer for amplifying the fliO gene until codon 85 | 1 | SJW1103 genomic DNA | pTSO27 | gtcattaaggatcctattaggcggtcacgcccagtac |
| 24 | 3`-fliO95 | Reverse PCR primer for amplifying the fliO gene until codon 95 | 1 | SJW1103 genomic DNA | pTSO28 | gtcattaaggatcctattacggcagggtgtgcagcag |
| 25 | 3`-fliO105 | Reverse PCR primer for amplifying the fliO gene until codon 105 | 1 | SJW1103 genomic DNA | pTSO29 | gtcattaaggatcctattaaacgggtgcttccgtgtc |
| 26 | 3`-fliO115 | Reverse PCR primer for amplifying the fliO gene until codon 115 | 1 | SJW1103 genomic DNA | pTSO30 | gtcattaaggatcctattacatcatgttctggaaatccgcc |
| 27 | 3`-fliO125-2 | Reverse PCR primer for amplifying the fliO gene | 1, 3 | SJW1103 genomic DNA | pTSO20; pTSO22 | gtcattaaggatcctattaggatctcccggaacgcttg |
| 56 | 5`-fliO6-Linear | Forward PCR primer to generate a linear plasmid PCR product at codons 6 and 7 of the fliO gene | 57 | pTSO17 | pTSO193; pTSO198 | acggtttctcagcccacc |
| 57 | 3`-fliO6-Linear | Reverse PCR primer to generate a linear plasmid PCR product at codons 6 and 7 of the fliO gene | 56 | pTSO17 | pTSO193; pTSO198 | ggcttctgtcttcatcatatg |
| 64 | 5`-fliO100-Linear | Forward PCR primer to generate a linear plasmid PCR product at codons 100 and 101 of the fliO gene | 65 | pTSO17 | pTSO195 | acggaagcacccgttgcc |
| 65 | 3`-fliO100-Linear | Reverse PCR primer to generate a linear plasmid PCR product at codons 100 and 101 of the fliO gene | 64 | pTSO17 | pTSO195 | gtcattctccgcgggcgg |
| 66 | 5`-fliO115-Linear | Forward PCR primer to generate a linear plasmid PCR product at codons 115 and 116 of the fliO gene | 67 | pTSO17 | pTSO196; pTSO201 | aagagcttactcaagcgttcc |
| 67 | 3`-fliO115-Linear | Reverse PCR primer to generate a linear plasmid PCR product at codons 115 and 116 of the fliO gene | 66 | pTSO17 | pTSO196; pTSO201 | catcatgttctggaaatccgcc |
| 79 | 5`-fliO6-phoA | Forward PCR primer to generate a phoA gene PCR product flanked with 15-bp homologous to the fliO gene | 80 | MG1655 genomic DNA | pTSO193 | atgaagacagaagcccggacaccagaaatgcctg |
| 80 | 3`- fliO6-phoA | Reverse PCR primer to generate a phoA gene PCR product flanked with 15-bp homologous to the fliO gene | 79 | MG1655 genomic DNA | pTSO193 | gggctgagaaaccgttttcagccccagagcggctttc |
| 87 | 5`-fliO100-phoA | Forward PCR primer to generate a phoA gene PCR product flanked with 15-bp homologous to the fliO gene | 88 | MG1655 genomic DNA | pTSO195 | cccgcggagaatgaccggacaccagaaatgcctg |
| 88 | 3`-fliO100-phoA | Reverse PCR primer to generate a phoA gene PCR product flanked with 15-bp homologous to the fliO gene | 87 | MG1655 genomic DNA | pTSO195 | aacgggtgcttccgttttcagccccagagcggctttc |
| 89 | 5`-fliO115-phoA | Forward PCR primer to generate a phoA gene PCR product flanked with 15-bp homologous to the fliO gene | 90 | MG1655 genomic DNA | pTSO196 | ttccagaacatgatgcggacaccagaaatgcctg |
| 90 | 3`-fliO115-phoA | Reverse PCR primer to generate a phoA gene PCR product flanked with 15-bp homologous to the fliO gene | 89 | MG1655 genomic DNA | pTSO196 | cttgagtaagctctttttcagccccagagcggctttc |
| 108 | 5`-fliO6-gfpuv | Forward PCR primer to generate a GFPuv gene PCR product flanked with 15-bp homologous to the fliO gene | 109 | pGFPuv | pTSO198 | atgaagacagaagccatgagtaaaggagaagaacttttcac |
| 109 | 3`-fliO6-gfpuv | Reverse PCR primer to generate a GFPuv gene PCR product flanked with 15-bp homologous to the fliO gene | 108 | pGFPuv | pTSO198 | gggctgagaaaccgttttgtagagctcatccatgccatg |
| 116 | 5`-fliO115-gfpuv | Forward PCR primer to generate a GFPuv gene PCR product flanked with 15-bp homologous to the fliO gene | 117 | pGFPuv | pTSO201 | ttccagaacatgatgatgagtaaaggagaagaacttttcac |
| 117 | 3`-fliO115-gfpuv | Reverse PCR primer to generate a GFPuv gene PCR product flanked with 15-bp homologous to the fliO gene | 116 | pGFPuv | pTSO201 | cttgagtaagctctttttgtagagctcatccatgccatg |
| 131 | 5`-fliO43-pTXB1 | Forward PCR primer for amplifying the fliO gene from codon 43 for cloning into plasmid pTXB1 by the In-fusion PCR cloning method | 132 | SJW1103 genomic DNA | pTSO133 | aaggagatatacatatgggattcgcgccaaaagg |
| 132 | 3`-fliO125-pTXB1 | Reverse PCR primer for amplifying the fliO gene with a 3` alanine codon for cloning into plasmid pTXB1 by the In-fusion PCR cloning method | 131 | SJW1103 genomic DNA | pTSO133 | atctcccgtgatgcaagcggatctcccggaacgctt |
| 165 | 5`-fliP-Nde1 | Forward PCR primer for amplifying the the fliP gene | 166 | SJW1103 genomic DNA | pTSP211 | gtattcggaaattccatatgcgccgtttgttattcctttc |
| 166 | 3`-fliP-BamH1 | Reverse PCR primer for amplifying the the fliP gene | 1; 165 | SJW1103 genomic DNA | pTSOP210;pTSP211 | gtcattaaggatccttactaactgtaaaagctttgggcc |
| 182 | 5`-fliO-6xHis | Site-directed mutagenesis primer for inserting codons for six histidine residues at the -3` end of the fliO gene | 183 | pTSO22 | pTSO220 | gcgttccgggagatcc**caccaccaccaccaccac**taataggatcctctag |
| 183 | 3`-fliO-6xHis | Site-directed mutagenesis primer for inserting codons for six histidine residues at the -3` end of the fliO gene | 182 | pTSO22 | pTSO220 | ctagaggatcctatta**gtggtggtggtggtggtg**ggatctcccggaacgc |
| 209 | 5`-fliO-Δ91 | Site-directed mutagenesis primer for deleting codon 91 of the fliO gene | 210 | pTSO17 | pTSO162 | gcagatcaacctgcacaccctgccgc |
| 210 | 3`-fliO-Δ91 | Site-directed mutagenesis primer for deleting codon 91 of the fliO gene | 209 | pTSO17 | pTSO162 | gcggcagggtgtgcaggttgatctgc |
| 230 | 5`-fliO-L91A | Site-directed mutagenesis primer for mutating codon 91 of the fliO gene to encode an alanine residue instead of a leucine residue | 231 | pTSO17 | pTSO239 | cgcagatcaacctg**gc**gcacaccctgccgc |
| 231 | 3`-fliO-L91A | Site-directed mutagenesis primer for mutating codon 91 of the fliO gene to encode an alanine residue instead of a leucine residue | 230 | pTSO17 | pTSO239 | gcggcagggtgtgc**gc**caggttgatctgcg |
| 325 | 5`-fliP-R143H | Site-directed mutagenesis primer for mutating codon 143 of the fliP gene to encode a histidine residue instead of an arginine residue | 326 | pTSOP210 | pTSOP259 | ctgcgccaaaccc**a**cgaagccgatctg |
| 326 | 3`-fliP-R143H | Site-directed mutagenesis primer for mutating codon 143 of the fliP gene to encode a histidine residue instead of an arginine residue | 325 | pTSOP210 | pTSOP259 | cagatcggcttcg**t**gggtttggcgcag |
| 327 | 5`-fliP-F190L | Site-directed mutagenesis primer for mutating codon 190 of the fliP gene to encode a leucine residue instead of a phenylalanine residue | 328 | pTSOP210 | pTSOP260 | cggcgtttcagatcggttttacgatt**c**tcatcccttttttg |
| 328 | 3`-fliP-F190L | Site-directed mutagenesis primer for mutating codon 190 of the fliP gene to encode a leucine residue instead of a phenylalanine residue | 327 | pTSOP210 | pTSOP260 | caaaaaagggatga**g**aatcgtaaaaccgatctgaaacgccg |
| 371 | 5`-BamH1-fliO | Forward primer for amplifying the fliO gene | 372 | pTSO20; pTSO22 | pTSPO325; pTSPO326 | ggcgc**ggatcc**aatttcacacaggaaacagcatatg |
| 372 | 3`-Pst1-fliO | Reverse primer for amplifying the fliO gene | 371 | pTSO20; pTSO22 | pTSPO325; pTSPO326 | gaaaa**ctgcag**atcctattaggatctcccgg |
| 375 | 5`-SigPep-FLAG-fliP | Site-directed mutagenesis primer for inserting codons for a FLAG-tag between codons 22 and 23 of the fliP gene | 376 | pTSP211 | pTSP324 | ccgccgccgctgcgcaa**gactacaaggacgacgatgacaaa**ctgccggggcttatcag |
| 376 | 3`-SigPep-FLAG-fliP | Site-directed mutagenesis primer for inserting codons for a FLAG-tag between codons 22 and 23 of the fliP gene | 375 | pTSP211 | pTSP324 | ctgataagccccggcag**tttgtcatcgtcgtccttgtagtc**ttgcgcagcggcggcgg |
