# Supplementary material for: FliO Regulation of FliP in the Formation of the Salmonella enterica Flagellum
Source: PLoS Genet. 2010 Sep 30;6(9):e1001143. doi: 10.1371/journal.pgen.1001143 (PMC2947984; doi:10.1371/journal.pgen.1001143)
Supplement: Text S1 — Supplementary materials and methods. (0.03 MB DOC) [file pgen.1001143.s009.doc]

Text S1

Materials and Methods

Alkaline phosphatase assays. Alkaline phosphatase activity was assayed according to an established method [1,2]. Salmonella enterica serovar Typhimurium was inoculated into 12-ml LB (with ampicillin if required) and grown with shaking to an OD_600_ of approximately 0.6 at 37^o^C. Cells (1.8-ml) were pelleted at 16,100-g for 5 minutes and re-suspended in 1.8-ml Tris-HCl (pH 8.0). To measure the OD_600_, 0.8-ml was taken. To the remaining 1-ml, 50-µl of 0.1% (w/v) SDS and 50-µl chloroform was added, cells were vortexed, and incubated at 30^o^C for 5 minutes. To the reactions, 100-µl of 0.4% (w/v) p-nitrophenyl phosphate was added and the reactions were incubated at 37^o^C. After the reaction had turned yellow or after a suitable time had elapsed 200-µl 1 M potassium hydrogen phosphate was added and the absorbance was measured at 420 nm and 550 nm. Units of phosphatase activity were calculated by the following formula:

1000 x (OD_420_ - [1.75 x OD_550_])/(time x OD_600_)

Bioinformatics. Protein alignments were made using MUSCLE [3]. Membrane topology predictions of aligned proteins were made using PolyPhobius [4]. For membrane topology predictions of individual, unaligned proteins the following programs were used: Phobius [5]; TMpred [6]; MEMSAT3 [7]; ConPred II [8]; TMHMM 2.0 [9]; HMMTOP 2.0 [10]; PHDhtm [11]; TMAP [12,13]; TopPred II [14]; and DAS [15].

1. Brickman E, Beckwith J (1975) Analysis of the regulation of Escherichia coli alkaline phosphatase synthesis using deletions and *φ*80 transducing phages. J Mol Biol 96: 307-316.
2. Michaelis S, Inouye H, Oliver D, Beckwith J (1983) Mutations that alter the signal sequence of alkaline phosphatase in Escherichia coli. J Bacteriol 154: 366-374.
3. Edgar RC (2004) MUSCLE: multiple sequence alignment with high accuracy and high throughput. Nucleic Acids Res 32: 1792-1797.
4. Käll L, Krogh A, Sonnhammer ELL (2005) An HMM posterior decoder for sequence feature prediction that includes homology information. Bioinformatics 21: i251-i257.
5. Käll L, Krogh A, Sonnhammer ELL (2004) A combined transmembrane topology and signal peptide prediction method. J Mol Biol 338: 1027-1036.
6. Hofmann K, Stoffel W (1993) TMBASE - A database of membrane spanning protein segments. Biol Chem Hoppe-Seyler 374: 166.
7. Jones DT (2007) Improving the accuracy of transmembrane protein topology prediction using evolutionary information. Bioinformatics 23: 538-544.
8. Arai M, Mitsuke H, Ikeda M, Xia J-X, Kikuchi T, et al. (2004) ConPred II: a consensus prediction method for obtaining transmembrane topology models with high reliability. Nucleic Acids Res 32: W390-W393.
9. Krogh A, Larsson B, von Heijne G, Sonnhammer ELL (2001) Predicting transmembrane protein topology with a hidden Markov model: application to complete genomes. J Mol Biol 305: 567-580.
10. Tusnády GE, Simon I (2001) The HMMTOP transmembrane topology prediction server. Bioinformatics 17: 849-850.
11. Rost B, Casadio R, Fariselli P, Sander C (1995) Transmembrane helices predicted at 95% accuracy. Protein Sci 4: 521-533.
12. Persson B, Argos P (1994) Prediction of transmembrane segments in proteins utilising multiple sequence alignments. J Mol Biol 237: 182-192.
13. Persson B, Argos P (1996) Topology prediction of membrane proteins. Protein Sci 5: 363-371.
14. Claros MG, von Heijne G (1994) TopPred II: an improved software for membrane protein structure predictions. Comput Appl Biosci 10: 685-686.
15. Cserzö M, Wallin E, Simon I, von Heijne G, Elofsson A (1997) Prediction of transmembrane *α*-helices in prokaryotic membrane proteins: the dense alignment surface method. Protein Eng 10: 673-676.
