# Supplementary material for: FliO Regulation of FliP in the Formation of the Salmonella enterica Flagellum
Source: PLoS Genet. 2010 Sep 30;6(9):e1001143. doi: 10.1371/journal.pgen.1001143 (PMC2947984; doi:10.1371/journal.pgen.1001143)
Supplement: Figure S1 — Alignment and predicted membrane topologies of FliO proteins from Gammaproteobacteria. SALTY = Salmonella enterica serovar Typhimurium; ECOLI = Escherichia coli K-12; ERWTA = Erwinia tasmaniensis; YEREN = Yersinia enterocolitica; XANCA = Xanthomonas campestris; VIBCH = Vibrio cholerae; and PSEAE = Pseudomonas aeruginosa. We have shown for Salmonella serovar Typhimurium FliO that the underlined residues in bold type could be truncated from the N-terminus or the C-terminus, and FliO still remained partially functional. We also identified residue leucine 91 (black bold-type) is important for the function of full-length Salmonella serovar Typhimurium FliO. The percentage score of the FliO homologues with Salmonella serovar Typhimurium FliO is indicated. (0.03 MB PDF) [file pgen.1001143.s001.pdf]

```
                SIGNAL PEPTIDE           PERIPLASM            MEMBRANE
SALTY       -----------------------MMKTEATVSQPTAPAGSP---LMQVSGALIGIIALIL 34
ECOLI       --------------------MNNHATVQSSAPVSAAP-------LLQVSGALIAIIALIL 33
ERWTA       ---------------------MNSTQQQSVTPQPAISTGSM---LTQVSGVLTAIIILIL 36
YEREN       -----------------------------------MPAGSV---LTQVGSVLGGILLLIL 22
XANCA       -----------MIGLLATAAPVAAKATGVGAQAPSSPS------LFGAVFALLLVLALIV 43
VIBCH       ---------------------MRKLAALFLIPVPGLAATSSQLDLATTLGSLLFVIALIL 39
PSEAE       MRRYLFVGFLPALASLSAPLCAAEGTTGAAAPTVGAASGAAAQ-LAQLVLGLGLVIGLIF 59
                                                        *      *     **

                                          CYTOPLASM
SALTY       AAAWVIKRMGFAPKGNSVRGLKVSASASLGPRERVVIVEVENARLVLGVTASQINLLHTL 94
ECOLI       AAAWLVKRLGFAPKRTGVNGLKISASASLGARERVVVVDVEDARLVLGVTAGQINLLHKL 93
ERWTA       AIAWLAKRFGFTARRGGSQQLNISASCQLGQRERVVIVDVDDARLVLGITAQQITHLHTL 96
YEREN       CAAWLVRRLGFAPQARNNKLLNVRASCQVGQRERVVIVEVDNTWLVLGVTAQNVTQLHTL 82
XANCA       GLGWLLKRMP-GSGFRSTEGLRVVTSLAVGAKERVVVVEVNGQQLLLGVTAGGISTLHTL 102
VIBCH       LLAWLLKRMR-VPAFGQQKGLSIIKQLPVGTRERVMIIQVGEEQYLIGVTTQSIQLLAKL 98
PSEAE       LLAWLVRRVQ-QAGPRGNRLIRTLASQPLGPRDRLVLVQVGEEQILLGLTPGRITPLHVL 118
               *   *                     *   *     *       * *      *  *

SALTY       P---PAENDTEAPVA----PPADFQNMMKSLL-KRS---------------GRS-- 125
ECOLI       P---PSAPTEEI-------PQTDFQSVMKNLL-KRS---------------GRS-- 121      67 %
ERWTA       PAK-PAQGSTDLPA-----APTDFRQILHSL--RKG---------------QGKKS 129      47 %
YEREN       PR--PPVDETDTALSADSGKPADFRQILNTYLLNKKSLLNKNSLLNKKSKPPEKSA 136      43 %
XANCA       PEPLPPTPAPTLPTFK---QLPNFAQLLAQKL-RKD---------------P---- 135      36 %
VIBCH       DN--PLIQEEL--------PATAFSNQLMQLLKKHE---------------TDSKT 129      29 %
PSEAE       KE--PVHLPDGEP------ATPEFAQRLLELL-NKD---------------PKGKP 150      28 %
                *                  *
```
